# Clinical retrospective analysis of peri-implant oral malignancies

**DOI:** 10.1186/s40729-024-00527-0

**Published:** 2024-02-06

**Authors:** Mi Hyun Seo, Mi Young Eo, Min Woo Park, Hoon Myoung, Jong Ho Lee, Soung Min Kim

**Affiliations:** Department of Oral and Maxillofacial Surgery, Dental Research Institute, School of Dentistry, Seoul National University, 101 Daehak-ro, Jongno-gu, Seoul, 110-768 South Korea

**Keywords:** Dental implant, Peri-implantitis, Peri-implant oral malignancy (PIOM), Squamous cell carcinoma (SCC), Galvanic current

## Abstract

**Purpose:**

Complications of implant prostheses have direct correlation with the increased use of implants for dental rehabilitation. In this study, we present cases of peri-implant oral malignancies (PIOM) around dental implants and a retrospective analysis of patients treated for PIOM.

**Methods:**

The retrospective analysis was performed with patients treated for PIOM at the Department of Oral and Maxillofacial Surgery of the Seoul National University Dental Hospital between 2006 and 2014. The patient records were thoroughly screened for previous medical issues, human papilloma virus infections, and other clinical data with a focus on relevant information such as localization, time from implant insertion to the development of the carcinoma, implant type and prosthetic rehabilitation.

**Results:**

Twenty-one patients were diagnosed with PIOM. The male-to-female ratio was 1.625. The mean age of the patients was 60.42 ± 9.35 years old. Three patients reported ongoing alcohol/tobacco consumption. Five patients had a history of previous oral cancer surgery or exhibited mucosal lesions. The time from implant placement until carcinoma diagnosis was 49.13 ± 33.63 months on average. Most PIOM patients (95.2%) were diagnosed with SCC. All patients had previously been treated for peri-implantitis. In 85.7% of the patients, prostheses were observed on the opposing teeth where PIOM occurred.

**Conclusion:**

Based on the review of these cases, it can be deduced that there is a possibility that implant treatment and galvanic currents between prosthesis may constitute an irritant and/or inflammatory cofactor which contributes to the formation and/or development of malignant tumors. Patients at potential risk may benefit from individualized recall intervals and careful evaluations.

**Supplementary Information:**

The online version contains supplementary material available at 10.1186/s40729-024-00527-0.

## Introduction

The known incidence of oral cavity cancers is the half ratio of all head and neck cancers, composing of 3 to 5% of all malignant tumors in humans. More than 90% of oral cancers are oral squamous cell carcinomas (OSCCs) whose etiology is multifactorial including tobacco use, alcohol consumption, human papilloma virus (HPV) infections, sun exposure especially in lip cancers, fat-rich diets, and dietary deficiencies in fruits and vegetables. There have been several changes in risk factors of OSCCs including being under the age of 40 and even in children, adolescents, and in women who do not possess any risk factors, compared with well-known risk factors such as OSCCs being typically found more in men than women over the age of 60 with habits of tobacco and alcohol use. Additionally, other less typical risk factors such as the existence of nutritional deficiencies, ionizing radiation exposure, immunosuppressants, chronic inflammation combined with periodontitis, and several irritant factors of dental and/or implant origin have been acknowledged as well.

Oral rehabilitation procedures using dental implants have been one of the best treatment options for fully or partially edentulous patients. The universal use of dental implants has increased the rate of several complications, one of which is the inflammatory process of alveolar bone and overlying soft tissues, namely peri-implantitis (PI).

The clinical appearances of PI are gingival erythema with edematous swelling, gingival hyperplasia or hypertrophy, and ulcers [1]. Occasionally, these manifestations require surgical biopsies for the differential diagnosis with malignant lesions [2, 3]. Even less on primary carcinomas in patients without a previous history of malignancy at the local or regional levels, OSCC cases with implants have increased as the number of implants placed have increased. To date, several articles on osseointegrated dental implants surrounding SCC cases have been published under the name of "mimicking or imitating peri-implantitis", "vicinity of dental implant" [4–6], "association or adjacent to dental implant" [7, 8], or "oral malignancy surrounding or after dental implants" [9, 10]. Therefore, we designated all kinds of malignancies surrounding dental implants with the term of peri-implant oral malignancy (PIOM).

More recently, a well-organized literature search by Kaplan et al. [11] showed that a total 47 cases of oral malignancies involving dental implants were published between 2000 and 2016 in 25 articles. This article showed a female predominance with a male/female ratio of 1:1.5 and a mean age of patients of 67.2 years. The mandible was involved in 89.4% cases, while the maxilla in 10.6% of cases. Forty cases at 85.1% were SCC, 29 cases at 61.7% were primary malignancies, and 4 cases at 8.5% were metastasis from distant tumors [11]. Unfortunately, detailed information regarding potential risk factors including history of smoking, alcohol abuse, fixture types, or individual prosthetic options, were not concluded due to the limitations of the review articles.

In the present study, our retrospective PIOM analyses were based upon our own clinical 21 cases including both the general significant factors and the specific implant factors, unlike other previous related studies. This is also the first study to report the statistical prognosis of more than 3 years of follow-up results in PIOM patients.

## Materials and methods

### Patient data acquisition

**Fig. 1:**
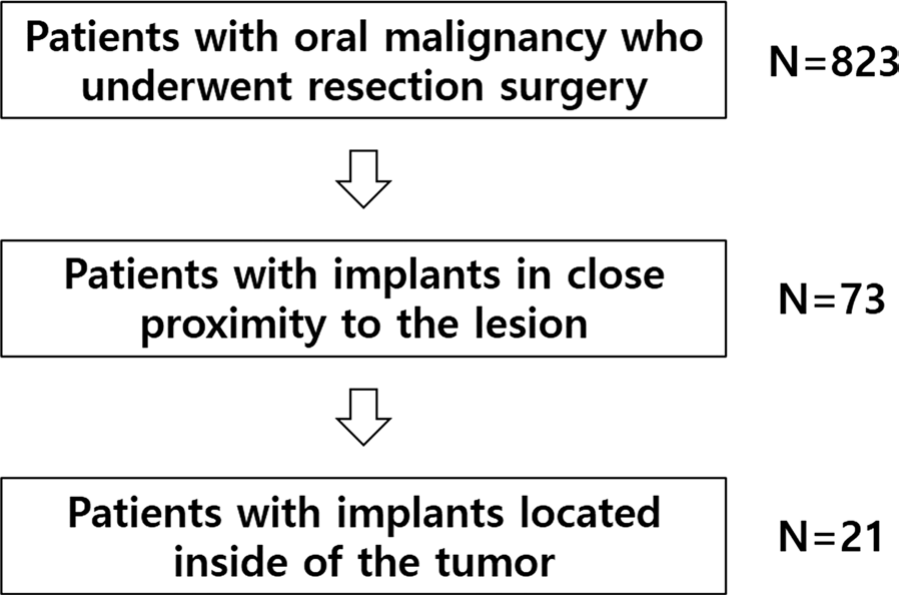
Peri-implant oral malignancy (PIOM) patient selection process. Among the 823 patients who underwent resection surgeries for oral cancer, patients with implants located in the center of the tumors were selected. N, patient number

Demographic information and clinical records were collected from 823 oral malignancy patients who underwent resection surgeries at the Seoul National University Dental Hospital (SNUDH), Korea. This study involved oral cancer patients between March 2006 and August 2014. Of these, only patients whose implants were located inside the tumors were included. Patients who had dental implants adjacent to malignant main masses were excluded from this study (Fig. 1). This study was conducted according to the guidelines of the Declaration of Helsinki and approved by the Institutional Review Board of Seoul National University (IRB No. S-D20170026).

### Clinical analysis

Clinical and radiographic features were collected from chart reviews, radiographs, and clinical photos. Basic demographic data, tumor location, features, implant location, cancer development period following implantation, implant surface and prosthesis type, combined bone graft procedures, presence of precancerous lesions, risk factors such as smoking and drinking, and oral hygiene maintenance were assessed. Marginal bone loss was determined using peri-implant view or panorama. The vertical distance from the implant abutment junction to the site of bone loss was measured, and in the panorama, the original length of the implant was used to compensate for the magnification rate.

### Histopathologic analysis

All histopathological interpretations were performed at the Department of Oral Pathology, SNUDH. The primary main mass was obtained in the operating room; a 5–10 mm^3^ specimen was extracted from the center portion of the main mass for HPV testing. According to the pathologic reports, we analyzed information about the primary tumor site, pathologic diagnosis, presence of bone involvement, degree of differentiation, and pathologic staging according to the 8th American Joint Committee on Cancer (AJCC) stage (Table 1).

**Table 1:** Demographic and clinical data of 21 patients with PIOM in this study

No.	Sex	Age	OH	S/A	PMH	Site	IS	Time to Ca (weeks)	PC	HPV	CA	Op. date	Dx	HF	pStage	BI	BG	ImpS	R	PM	OccP	OccPM
1	M	44	Moderate	–	n/s	Lower	45,46,47	48	–	n.c	E	2008-02-21	SCC	Well	IV	Pr	-	TiO_2_	Sc	Gold	P	PFM
2	F	48	Poor	–	HTN	Lower	43,44	9	Previous SCC	n.c	E	2008-06-23	SCC	Poor	IV	Pr	Auto	SLA	Sc	Gold	None	–
3	M	54	Moderate	S + A	n/s	Lower	46,47	10	–	n.c	E	2008-12-22	SCC	Moderate	IV	Pr	Auto	RBM	Sc	Gold	P/implant	PFM
4	F	56	Moderate	–	Metal allergy	Lower	45,46,47,48	41	–	n.c	EU	2009-02-16	SCC	Well	IV	Pr	-	SLA	Sc,C	Gold/Sc,PFM/C	None	–
5	M	72	Poor	–	LC	Lower	32,33,35,36,37	14	–	n.c	EU	2009-10-22	SCC	Well	IV	Pr	-	RBM	C	PFM	P	PFM
6	M	61	Moderate	–	n/s	Lower	34,35,36,37	90	–	n.c	U	2010-02-22	SCC	Well	IV	Pr	-	TPS	C,Sc	PFM/C,Gold/Sc	P/implant	Gold
7	F	69	Moderate	–	Osteoporosis	Upper	13,14,15,16,17	24	–	n.c	EU	2010-08-05	SCC	Well	IV	Pr	-	HA blast and acid wash	C	PFM	P/implant	PFM
8	F	68	Good	–	Aspirin	Upper	16,17	–	–	n.c	U	2011-01-06	SCC	Moderate	IV	Pr	-	TiO_2_	C	PFM	P/implant	PFM
9	M	61	Moderate	S + A	HTN	Upper	26,27	120	–	None	EU	2011-03-31	SCC	Well	II	Ab	Xeno	HA coated	Sc	PFM	P/inlay	Gold
10	M	59	Moderate	–	n/s	Lower	34,35	–	–	None	EU	2011-04-14	SCC	Well	I	Ab	-	RBM	C	PFM	P	PFM
11	M	63	Moderate	–	Drug allergy	Lower	45,46,47	60	–	None	EU	2011-10-11	SCC	Well	IV	Pr	-	SLA	Sc	PFM	P/implant	PFM
12	F	64	Moderate	–	n/s	Upper	16	17	–	n.c	EU	2012-03-16	SCC	Well	IV	Pr	Xeno	HA blast and acid wash	C	PFM	P/implant	PFM
13	F	76	Poor	–	HTN, TB Hx, HL	Lower	45,46,47	100, 74	OLP	None	E	2012-08-13	SCC	Well	I	Ab	Xeno	Ti-unite	C	PFM	P	PFM
14	M	58	Moderate	–	n/s	Lower	36,37	58	Previous SCC	n.c	EU	2012-12-24	SCC	Well	IV	Pr	Syn	RBM	Sc	Gold	P/implant	PFM
15	M	63	Moderate	S	GC, DM	Upper	26,27	24	–	n.c	E	2013-03-12	SCC	Poor	II	Ab	Xeno	HA coated	C	PFM	P/inlay	Gold
16	F	58	Moderate	–	DM	Lower	35,36	84	–	None	E	2013-04-15	SCC	Well	IV	Pr	Auto	SLA	C	PFM	None	–
17	F	40	Poor	–	n/s	Upper	23,25	59	Candidiasis	None	E	2013-04-18	SCC	Well	II	Ab	Xeno	Ti-unite (Ti-O2)	Sc	Gold	P	PFM
18	M	71	Moderate	–	HTN	Lower	36,37	79	–	None	EU	2014-04-22	SCC	Well	IV	Pr	Xeno	SLA	C	PFM	P	Gold
19	M	51	Moderate	–	n/s	Upper	24,26, 27	–	–	n.c	E	2010-12-17	Melanoma	Well	I	Ab	-	SLA	C	PFM	P	Gold
20	M	63	Moderate	–	HTN	Upper	26	–	–	None	E	2017-07-24	SCC	Well	IV	Pr	-	TiUnite	C	Gold	P	PFM
21	M	70	Poor	–	DM	Lower	45,46,47	–	Previous SCC	None	EU	2017-08-14 2018-01-15	SCC	Well	IV	Pr	Allo	TiUnite	C	PFM	P	PFM

No.: number; OH: oral hygiene; S/A: smoking or alcohol; PMH: past medical history; IS: implant site; PC: precancerous lesion; HPV: human papilloma virus detected through Microarray; CA: clinical appearance; Op.: operation; Dx: diagnosis; HF: histopathologic finding; pStage: pathologic staging; BI: bone involvement; BG: bone graft history; ImpS: implant surface treatment; RS: retention; PM: prosthesis material; OccP: occluding prosthesis presence; OccPM: occluding prosthesis material

M: male; F: female; n/s: non-specific; HTN: hypertension; LC: liver cirrhosis; Mx: medications; TB Hx: pulmonary tuberculosis history; HL: hyperlipidemia; GC: gastric cancer; DM: diabetes mellitus; SCC: squamous cell carcinoma; OLP: oral lichen planus; n.c: not checked; E: exophytic; U: ulcerated; EU: exophytic + ulcerated; VC: verrucous carcinoma; Pr: presence; Ab: absence; Auto: autogenous bone graft; Allo: allogenic bone graft; Xeno: xenogenic bone graft; Syn: synthetic bone graft; SLA: sand blasted and acid-etched; RBM: resorbable blast media; TPS: titanium plasma spray; HA: hydroxyapatite; Sc: screw type; C: cemented type; P: prosthesis; PFM: porcelain fused to metal prosthesis

## Results

### Patient characteristics

In this retrospective study, we scanned a total of 823 patients who underwent malignant mass resections in our department during the period from March 2006 to August 2014. There were 454 males and 369 females with a male–female ratio of 1.23:1. The mean age of all the patients was 59.4 years.

**Fig. 2:**
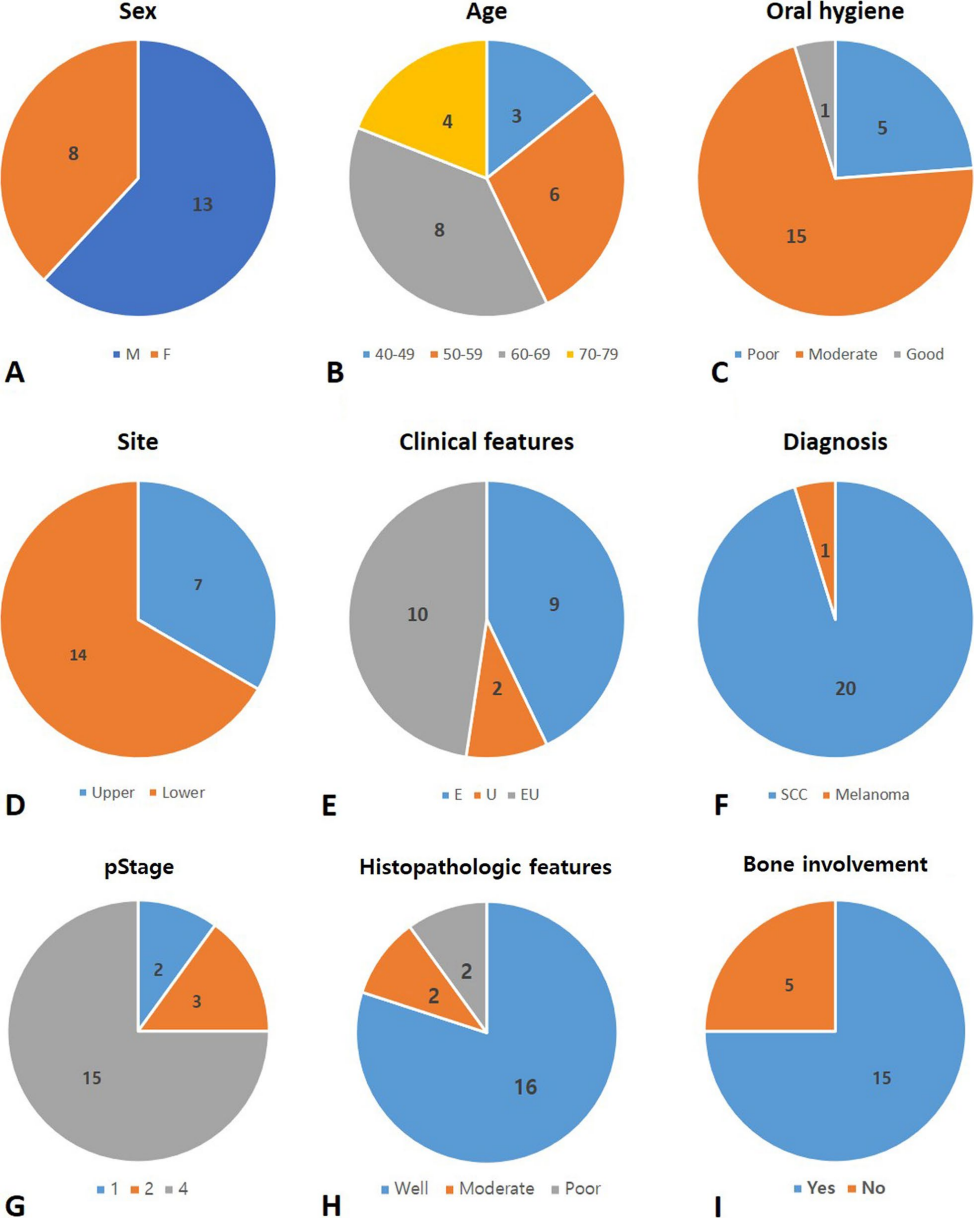
Pie charts that summarize the demographics, risk factors, clinical features, and pathological findings of the PIOM patients. The incidence of carcinomas next to dental implants is low but may attain clinical relevance with increasing dental implant treatment. For pathological staging (pStage), histological features, and bone involvement, only patients diagnosed with oral squamous cell carcinomas (OSCCs) were included. M, male; F, female; E, exophytic; U, ulcerative; SCC, squamous cell carcinoma; pStage, pathological staging

Seventy-three patients who had implant fixtures related or adjacent to the malignant masses were enrolled in additional clinical and radiograph data collecting stages of the study. Twenty-one patients who had one or many implant fixtures located inside of the tumor main mass were eligible for the study after using the inclusion and exclusion criteria (Fig. 1). This PIOM study population made up 2.55% of the malignant surgery cases in our hospital in a nearly 9-year period. Table 1 shows the patient demographic data of the PIOM cases. The PIOM cases consisted of 13 males and 8 females, having a ratio of 1.625:1 with an average age 60.43 ± 9.35 and range of 40–76 years (male: 60.77, female: 59.88). There were five non-survival patients until the conducting time of this study. The 5-year survival rate was 76.2% (Table 1, Fig. 2A, B).

### Patient risk factors and habitual factors

Three patients were previously treated with OSCC in the tongue and lower gingiva accompanied by postoperative irradiation, and one patient underwent a partial gastrectomy 5 years ago. The other two patients had previously been routinely checked for oral lichen planus and candidiasis. There were only three patients who had the habit of smoking with two of them found to be drinking as well. Nine patients were HPV negative and 12 patients were unidentified for HPV infection. Five patients exhibited poor oral hygiene, 15 patients demonstrated moderate hygiene status, and only one patient showed good oral hygiene (Table 1, Fig. 2C).

### Clinicopathological characteristics of PIOM

Regarding position distribution, there were 14 mandible cases (66.7%) with 39 fixtures (70.9%), higher than the 7 maxilla cases with 16 fixtures (29.1%). Among the 55 implants, 50 fixtures were located in the posterior region (90.9%). There were four cases (Patient No. 02, 05, 07 and 17) with a total of 5 fixtures that had lesions related to the anterior region (canine region) (Fig. 2D).

**Fig. 3:**
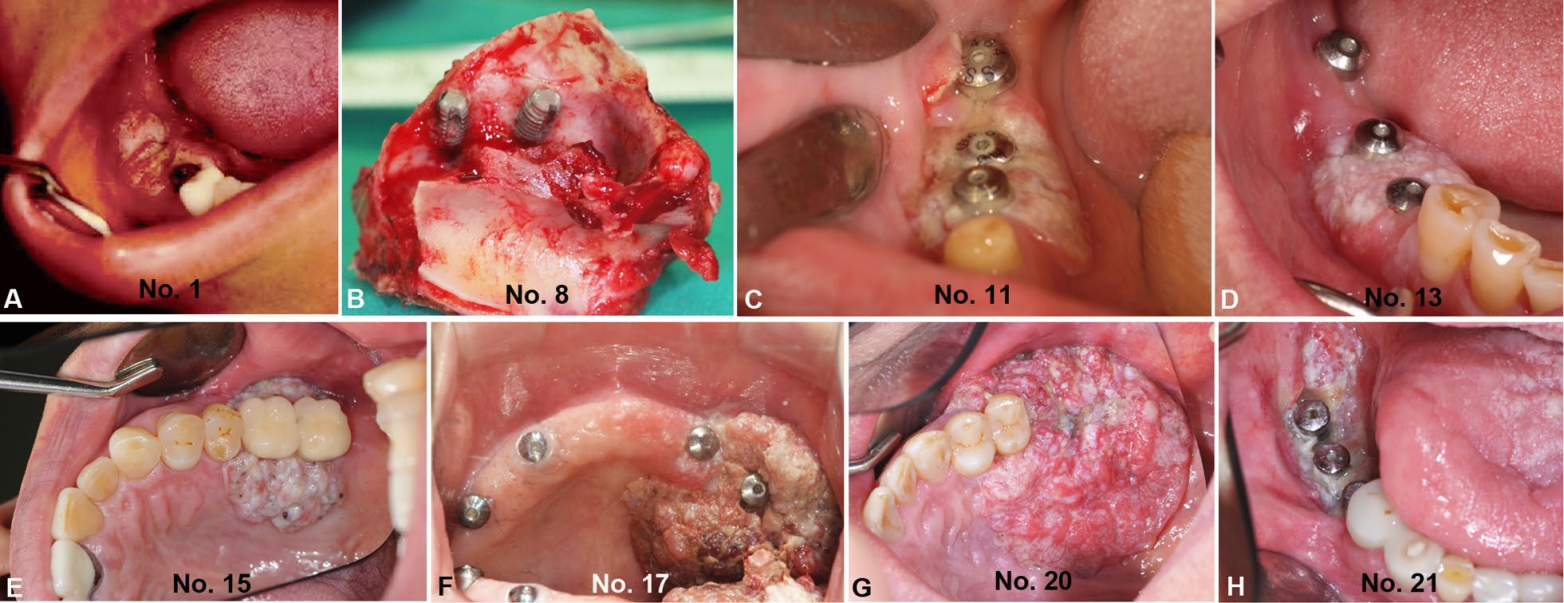
Clinical pictures of the patients diagnosed with PIOM. Most of the lesions exhibited an exophytic appearance

**Fig. 4:**
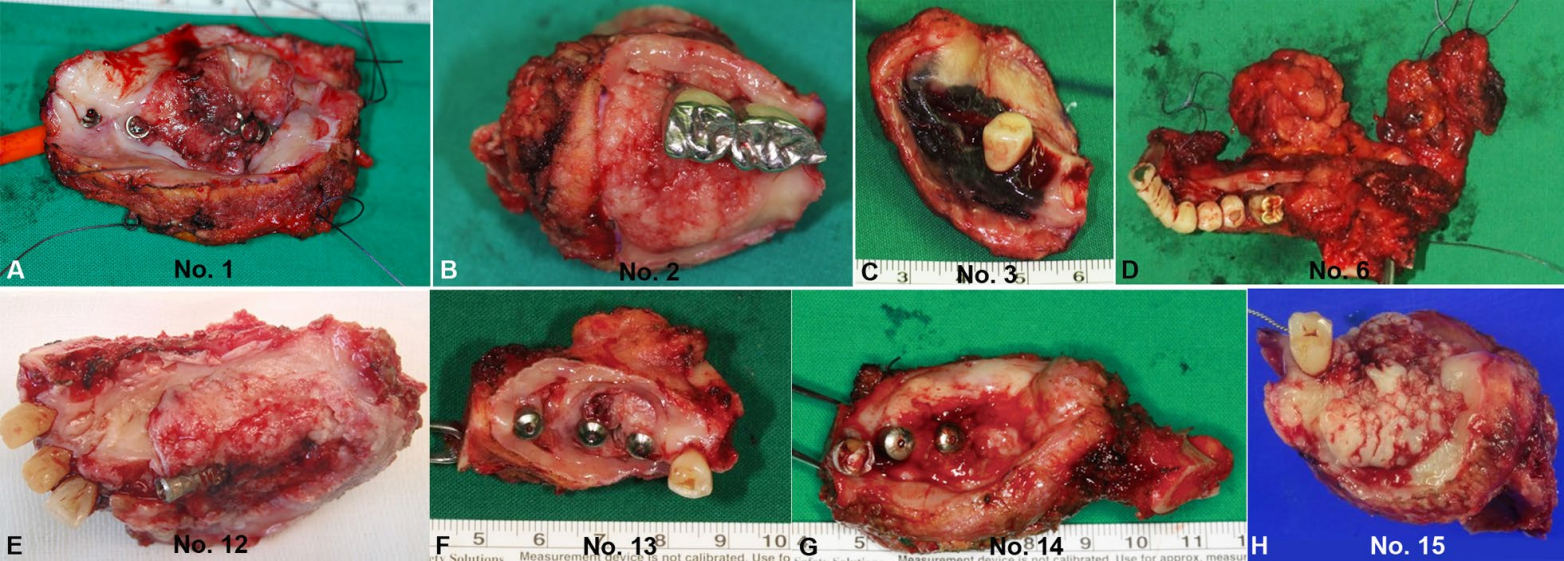
Clinical pictures of the resected tumors from the PIOM patients. Dental implants are included in the center of the masses

The clinical appearance was classified as exophytic, ulcerated, or a combination of exophytic and ulceration. Most of the cases were exophytic (*n* = 19), of which 10 cases exhibited both exophytic and ulcerated appearances, while nine cases displayed only exophytic features. There were only two cases recorded as ulcerated types (Figs. 2E, 3). The morphologies of the excised masses are shown in Fig. 4 with the center of the masses containing dental implants. All 21 patients received treatment for PI before the diagnosis of cancer. Marginal bone loss was identified in 13 patients from a panoramic or periapical X-ray (Additional file 1). In plain X-ray, marginal bone loss was observed in 61.9% of the overall patients. This is attributed to a high incidence of buccal bone loss among patients. Until a certain extent of buccal bone loss occurs, there may be no alteration in the inter-implant bone levels at the mesial and distal aspects of the implant fixtures.

**Fig. 5:**
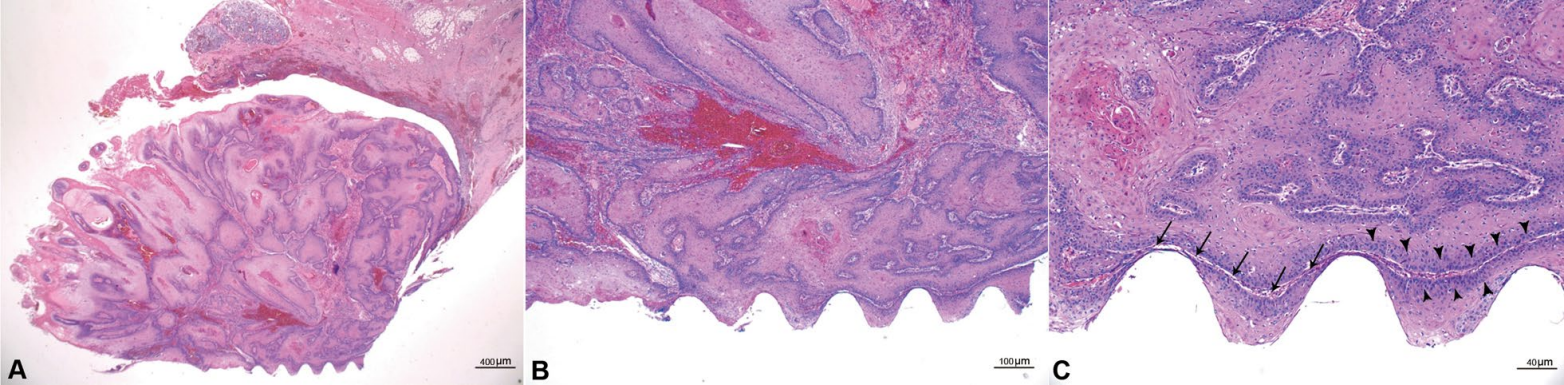
Representative histological pictures showing the key features of PIOM. It shows the aspect of outward growth from the center of the implant. **A** ×40 magnification, H &E, **B** ×100 magnification, H &E, **C** ×400 magnification, H&E, arrows; boundary between the tumor and implant, arrowheads; tumor cells clustered along the implant

Most of the PIOM patients were diagnosed with SCC, and only one patient was diagnosed with melanoma. The pathologic stages were identified in the OSCC patients through pathologic reports after surgery. Stage IV, II, and I patients were 15, 3, and 2 in number. Seventy-five percent of OSCC patients were observed at the advanced stage. Bone involvement was present in 15 cases. SCC differentiation grades were well-differentiated in 16 cases, moderately differentiated in two cases, and poorly differentiated in two cases. Most of the PIOM cases were well-differentiated (80%) (Fig. 2F–I). In most aspects of PIOM, outward growth around the dental implants was observed, and tumor cells were densely concentrated at the interface between the implant and the tumor in the histological findings (Fig. 5).

### Dental implications: grafted bone materials, implant fixtures, and prostheses

Out of the 21 PIOM patients, 11 patients had the record of receiving bone grafting prior to or at the same time with implant installation. The bone graft materials used varied, including autogenous bone grafts (*n* = 3, of which there was one iliac bone graft case), allogenic bone grafts (*n* = 1), xenogenic bone grafts (*n* = 6), and synthetic bone grafts (*n* = 1).

Among the 21 PIOM patients, three patients underwent implant placement surgery at SNUDH, while the other 18 patients had implants placed in different hospitals or clinics. Among the 55 implant fixtures, 18 of them were cylindered type implants. The surface treatment of the implant fixtures was diverse, including Ti Unite (four cases with 9 fixtures), sandblast and acid etching (six cases with 16 fixtures), HA-coated (two cases with four fixtures), resorbable blast media (four cases with 11 fixtures), titanium plasma spray (one case with four fixtures), hydroxyapatite blast and acid wash (two cases with six fixtures), and TiO_2_ (two cases with five fixtures).

**Fig. 6:**
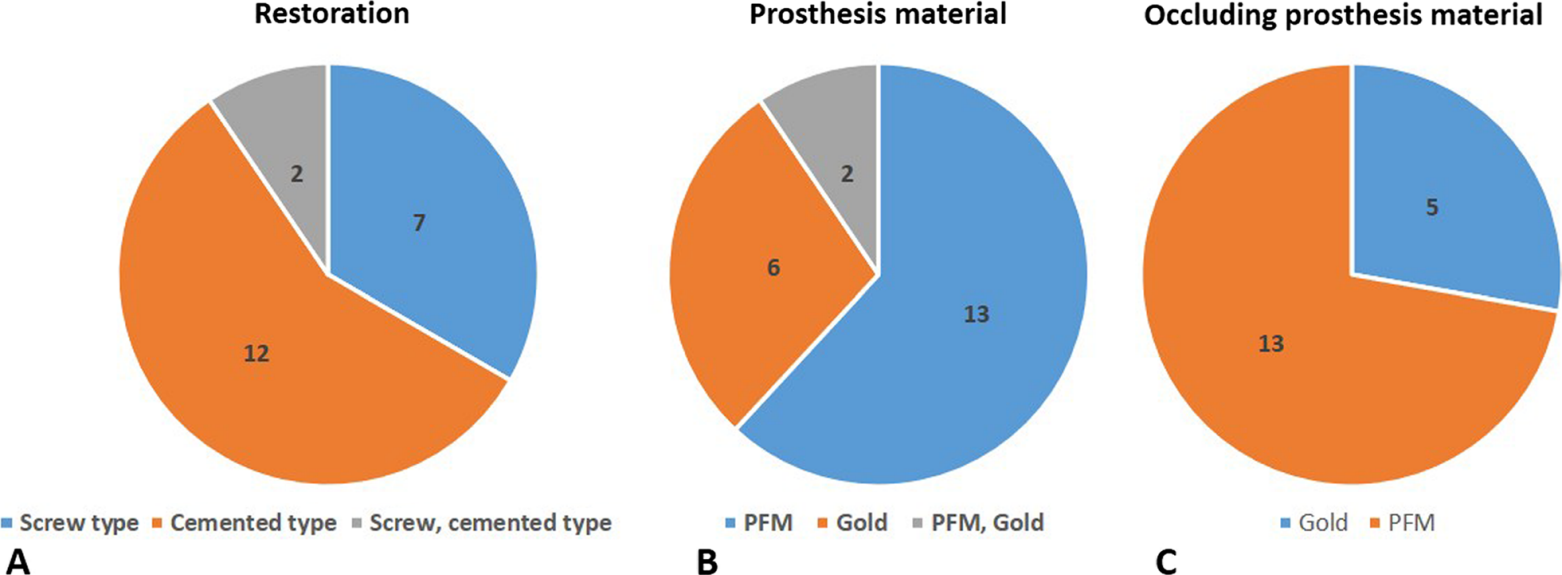
Summary of dental features with a focus on implants included in PIOM and opposing prostheses

**Fig. 7:**
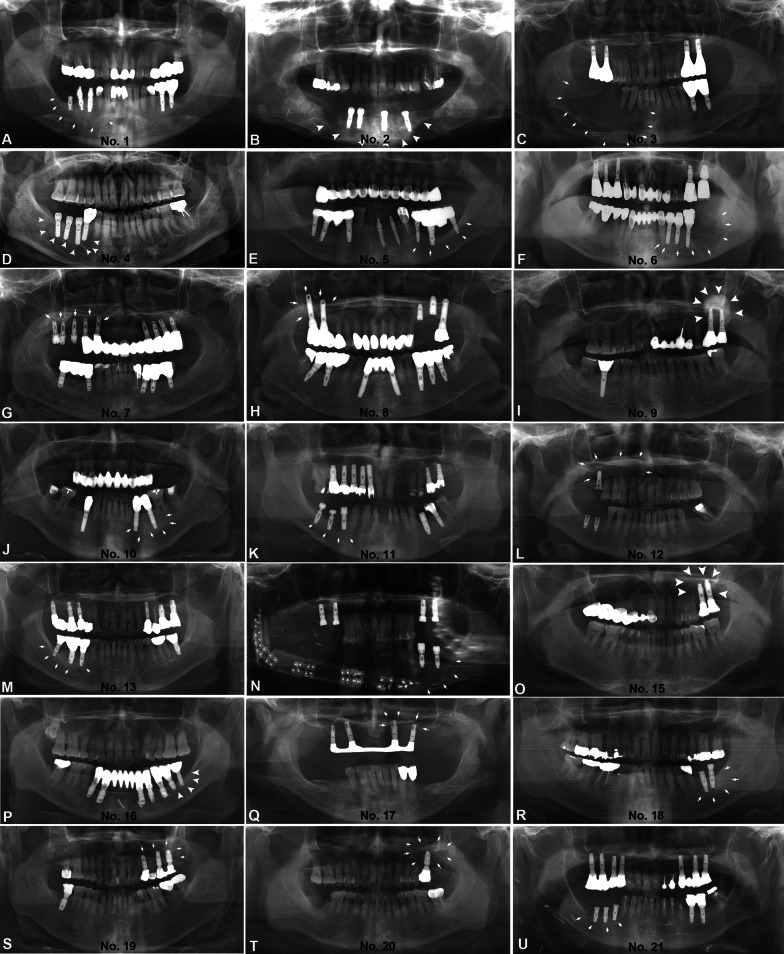
Panoramic pictures before surgery of the patients included in this PIOM study. White arrows, arrowheads; tumor margins

Twenty patients had fixed prosthetics and one patient had a removable prosthesis (bar-retained overdenture). Thirteen patients had porcelain fused to metal prostheses (PFM), 6 patients had gold prostheses, and 2 patients had both PFM and gold prostheses. Opposing occlusion prostheses was presented in 18 cases, of which 9 cases were prosthesis/prosthesis occlusion, two cases were prosthesis/inlay occlusion, and seven cases were prosthesis/prosthesis on implants. The occlusal opposing prostheses or inlays were fabricated from PFM (*n* = 13) or gold (*n* = 5) (Table 1, Fig. 6). The sites of the PIOM and the prosthesis of the opposing teeth were obtained by referring to clinical photos and dental panoramas. The overall panoramic views are shown in Fig. 7. Most of the patients had prostheses that occluded the implants. The average duration from implant insertion to the diagnosis of malignancy was 49.13 ± 33.63 months (range 9–120 months). A total of 55 fixtures with their connected prostheses were directly in contact with the malignant lesions.

### Prognosis analysis

In the Kaplan–Meier analysis of 5-year survival for all-cause mortality, the patients diagnosed with PIOM exhibited a 76.2% 5-year survival rate. OSCC patients except for one melanoma patient demonstrated a 5-year survival rate of 80.0%.

## Discussion

From the several articles regarding case reports and related review articles, the possible contributing factors of relevant PIOM could be summarized as: (1) dental implant corrosion and possible association between corrosion products and SCC; (2) the possible association between particulate titanium and SCC; (3) migration of malignant cells through the implant surrounding sulcus; and (4) the hypothesized carcinogenic effect of sustained metallic ion release after implant placement [7, 11, 12].

Commercial pure titanium (CP-Ti) or a Ti6Al4V alloy could deteriorate from the surrounding medium attachments causing electrochemical or galvanic currents, leading to the possible association between corrosion product release and SCC. This hypothesis could be accepted especially in failing or failed implants, which may occur in many cases of PI [13]. Titanium ions are well-known to be one of the most inert metallic ions with a very low corrosion rate of 0.003 μA/cm^2^ [14]. Particulated implant debris could result in inflammation round orthopedic implants. Therefore, inflammatory factors such as eicosanoids, collagenase, interleukin-1, and prostaglandin E-2 could lead to implant bursitis and bony resorption [15, 16]. Dental implant placement is a type of entrance for malignant cells and this migration through implants contacting the gingival sulcus has also been suggested for PIOM.

From these three hypotheses, the carcinogenic effect of metallic ion release has been suggested in a more detailed way and is divided into three separate issues including: (1) the carcinogenicity of the metallic ions; (2) exposure level to the patient; and (3) incidence of cancer in patients treated with implants [17–19]. Adding to the corrosive carcinogenicity of titanium ions, the exposure level to the patient would be dependent on the surface area of the implant and exposed time duration. The number of implants should be considered for these suggestions, and although it is difficult to establish a threshold of how many implants would constitute a significant total surface area, the only conceivable situation where a significant surface area would exist would be in patients with multiple implants. Unlike several case reports which were related to only a single implant, our retrospective results revealed that only two cases of patients No. 12 and 20 possessed a PIOM surrounding the upper first molar. Otherwise, the 19 other cases of PIOM were all multiple implant cases between two to five implants (Table 1). Our results could support the hypothesis that PIOM would be observed relating to patients with multiple implants where the exposed surface area and incidence would be higher. Unfortunately, the direct carcinogenic role of dental implants has yet to be established, but just hypothesized or proposed by several clinicians.

Although dental implants have seen tremendous clinical success over the last few decades, there are some worrying reports in literature describing SCC in close association with dental implants. This article also provides a critical assessment of the published literature relating to the presence of carcinomas in association with dental implants, analyzed previously published and hypothesized carcinogenic responses to an implant, and attempted to come to a conclusion regarding the plausibility and clinical risks for cancer formation in association with dental implants [2, 4, 7, 9, 20, 21].

An unusual case of an SCC noted in close proximity to a dental implant is also presented. A systematic search was conducted using Medline (PubMed), Cochrane Database, and Google Scholar with the search terms "cancer", "squamous cell carcinoma", "dental implant", "SCC", "peri-implantitis", "oral cancer", and "implantology" using multiple combinations with the boolean operators "or" and "and". The search was not limited to dental literature—orthopedic and biomedical literature were also included.

The results were then screen to locate relevant articles. In total, 14 previous published reports were found, where 24 dental implants were reported to be associated with SCC. Not all the reported patients had a history of cancer, but contributory factors such as smoking were observed. An analysis of the biological plausibility of previously proposed carcinogenic mechanisms such as corrosion, metallic ion release, and particulate debris did not support the etiologic role of dental implants in cancer development. PI must be assessed cautiously in patients receiving implants who have a previous history of cancer. Dental implants are a safe treatment modality based on the published data, and any changes in the surgical protocol are not mandated.

The sex ratio of PIOM was balanced as male 1.625 to female 1.0, which could be compared with the general OSCC ratio of 1.43 to 1.0. Individual living patterns including habits, occupation, foods, and oral hygiene must be supported for this gender ratio difference.

Both alcohol and tobacco consumption were observed in three patients who exhibited very low proportions of classical risk factors of OSCC. As previous report of upregulation of the interferon-γ and nuclear factor kappa B in risk factor studies of nonsmokers and nondrinkers demonstrated [9, 22], different carcinogenesis of PIOM could be suggested in the point of typical “smoking and alcohol” risk patterns. Among our cases, three patients had a history of previous cancer treatment. This is also a lower proportion compared with other previous reports.

The most common clinical feature was an exophytic mass with ulceration around the implants, followed by an exophytic mass without ulceration. All patients were treated for PI before diagnosis, and routine dental radiography was not a valuable criterion for differential diagnosis. In panoramic or periapical view, the incidence of observed marginal bone loss was 61.9% of the total cases. Buccal bone loss was frequently noted in the CT, it may not manifest in the bone levels at the peri-implant fixture on the distal and proximal side. Furthermore, it is believed that PIOM is characterized by an exophytic growth pattern, influencing clinical features. Additionally, when bone destruction around implants is irregularly evident on CT scans, it may not be apparent in plain film imaging. The use of plain film for measurements has limitations in reflecting the level of marginal bone loss, and CT scans are challenging due to metal artifacts. In this study, only 61.9% demonstrated marginal bone loss, but all patients had a history of previous diagnosis and treatment for PI including debridement.

In the differentiation of tumors, well-differentiated SCC was the most common type. In patients excluding one case of melanoma, the 5-year survival rate was 80% with 75% of the patients being stage IV, and patients with bone involvement accounted for 75% of advanced stage surgery, which exhibited good prognoses. 85.71% of PIOM patients had prostheses containing metal components and not natural teeth. 72.2% of opposing prostheses were made of PFM. Although the PIOM patient group have a relatively low risk factors, we carefully speculate that the galvanic currents formed by contacting various types of metals may have contributed to the occurrence of PIOM. Additional research is needed to support this thesis.

There is a debate regarding whether malignant changes occur in PI or if the manifestation of PIOM presents signs similar to PI. The question arises regarding whether the existence of dental implants poses a risk for malignancy. PI is characterized by persistent and prolonged inflammation. Chronic inflammation has been associated with malignant transformations in various types of cancers, as seen, for example, in patients with Crohn's disease developing colon cancer [11]. If inflammation persist, it can have sufficient potential to induce cellular proliferation and prolong cellular survival by activating the oncogenes and inactivating tumor suppressor genes, which would produce genetic instability and a greater risk of having cancers [23]. The concentration of titanium particles increases significantly at PI sites. However, it remains unclear whether this phenomenon also trigger inflammation and bone resorption [24]. As of now, there is no established association between the occurrence of dental implants and OSCC [23, 25].

Based on the review of these cases, it can be deduced that there is a possibility that implants and galvanic currents between different prostheses may constitute an irritant and/or inflammatory cofactor contributing to the formation and/or development of OSCC. Basic research is needed to establish a clear cause–effect relationship. The incidence of carcinomas next to dental implants is low but may attain clinical relevance with increasing dental implant treatment. Patients at risk potentially profit from individualized recall intervals and careful evaluations.

It is essential to perform a delicate oral cavity examination before implant treatment, and the patient’s risk factors of SCC must also be managed and controlled with strict follow-up protocols. Appropriate regular check-ups for the patients with risk factors must be conducted, and histopathologic biopsy examinations should be performed as soon as possible on any questionable lesions. Although frequent biopsies of every case of PI would not be justified, clinical PIOM resembling cases including hyperplastic papillomatous gingival growth or whitish alveolus covering the gingiva should be promptly biopsied promptly. During this strict periodic follow-up period, high-resolution self-photos with individual mobile phones could be an alternative for frequent visits to the clinics. Fortunately, most of our represented PIOM cases were recognized clinically, thus if patients had been educated properly, each patient could regularly send his or her intraoral photos to the specialized clinics. However, in order to elucidate the precise pathogenic mechanism involved in each of these disease processes, additional molecular genetic investigations should be performed.

## Conclusion

The role of osseointegrated implants in the formation of SCC is not well-established. Most frequent carcinoma associated with dental implants, named as PIOM, occur in the form of PI, exhibiting exophytic masses with or without ulcerations. Regular check-ups are mandatory for the early detection of the development of PIOM, especially in the patients having any types of intraoral prosthesis.

### Supplementary Information

**Additional file 1.** Marginal bone loss demonstrated in the panoramic or peri-implant view.

## Data Availability

The datasets used and/or analyzed during the current study are available from the corresponding author on reasonable request.
