# Supplementary material for: Clinical retrospective analysis of peri-implant oral malignancies
Source: Int J Implant Dent. 2024 Feb 6;10:5. doi: 10.1186/s40729-024-00527-0 (PMC10847072; doi:10.1186/s40729-024-00527-0)
Supplement: Supplementary file 1 — Additional file 1. Marginal bone loss demonstrated in the panoramic or peri-implant view. [file 40729_2024_527_MOESM1_ESM.docx]

Additional file 1. Marginal bone loss demonstrated in the panoramic or peri-implant view

| **Patient No.** | **Implant site** | **Mesial bone loss (mm)** | **Distal bone loss (mm)** |
| --- | --- | --- | --- |
| **1** | 45 | 0 | 0 |
|  | 46 | 1.9 | 2.7 |
|  | 47 | 3.7 | 4.0 |
| **2** | 43 | 0 | 0 |
|  | 44 | 0 | 0 |
| **3** | 46 | _ | _ |
|  | 47 | _ | _ |
| **4** | 45 | 0 | 0 |
|  | 46 | 0 | 0 |
|  | 47 | 0 | 0 |
|  | 48 | 0 | 0 |
| **5** | 32 | 0 | 0 |
|  | 33 | 0 | 0 |
|  | 35 | 2.3 | 3.3 |
|  | 36 | 1.5 | 3.3 |
|  | 37 | 1.9 | 1.9 |
| **6** | 34 | 0 | 0 |
|  | 35 | 1.1 | 1.1 |
|  | 36 | 1.1 | 5.0 |
|  | 37 | _ | _ |
| **7** | 13 | 0 | 0 |
|  | 14 | 0 | 0 |
|  | 15 | 0 | 0 |
|  | 16 | 5.8 | 4.7 |
|  | 17 | 4.0 | 6.8 |
| **8** | 16 | 1.2 | 3.1 |
|  | 17 | 2.7 | 2.7 |
| **9** | 26 | 0 | 0 |
|  | 27 | 0 | 0 |
| **10** | 34 | 0 | 0 |
|  | 35 | 0 | 0 |
| **11** | 45 | 0 | 0 |
|  | 46 | 0 | 0 |
|  | 47 | 0 | 0 |
| **12** | 16 | 3.6 | 6.9 |
| **13** | 45 | 0.8 | 2.9 |
|  | 46 | 2.6 | 0.9 |
|  | 47 | 4.2 | 2.3 |
| **14** | 36 | 0 | 1.9 |
|  | 37 | 3.9 | 4.3 |
| **15** | 26 | 2.9 | 3.4 |
|  | 27 | 3.6 | 1.1 |
| **16** | 35 | 3.7 | 2.2 |
|  | 36 | 2.1 | 0.8 |
| **17** | 13 | 1.0 | 1.1 |
|  | 15 | 6.0 | 6.4 |
|  | 23 | 1.2 | 2.1 |
|  | 25 | 3.7 | 4.0 |
| **18** | 36 | 1.1 | 2.5 |
|  | 37 | 3.5 | 2.6 |
| **19** | 24 | 0 | 0 |
|  | 26 | 0 | 0 |
|  | 27 | 0 | 0 |
| **20** | 26 | 0 | 0 |
| **21** | 45 | 0 | 0 |
|  | 46 | 0 | 0 |
|  | 47 | 0 | 0 |

- : unchecked data
